# Do orthodontists consider endodontic complications in their orthodontic management of teeth with a history of dental trauma? A vignette survey

**DOI:** 10.1186/s12903-024-05286-3

**Published:** 2025-01-16

**Authors:** Kinda Awad, Ammar Al Hourani, Dariusz Kasperek, Fadi Jarad, Sondos Albadri

**Affiliations:** University of Liverpool, Liverpool, United Kingdom

**Keywords:** Trauma, Orthodontics, Endodontics, Vignette

## Abstract

**Background:**

Dental trauma is a frequent injury seen commonly in young children. There is a link between malocclusion and the incidence of traumatic dental injuries. Orthodontic therapy has been suggested as a preventative measure in correcting unfavourable malocclusions and potentially avoiding traumatic dental injuries. To date, it is poorly understood if the complications reported following traumatic dental injuries are amplified during and following orthodontic treatment. The aim of this study was to evaluate whether orthodontists considered the endodontic implications associated with the orthodontic treatment of teeth with a history of dental trauma.

**Method:**

A mixed methods vignette survey was designed, piloted, and distributed online to UK registered specialist orthodontists. The survey was split into three parts and consisted of three vignette clinical scenarios with open and closed questions.

**Results:**

A total of 76 orthodontists responded from the United Kingdom. Of the participants, 46% (*n* = 35) of the orthodontists felt they had insufficient training in dental trauma and 42% (*n* = 32) lacked confidence in the treatment of traumatic injuries. The study participants reported non-standardised pre- treatment examination, limited dental trauma experience and insufficient training. In addition, 32% (*n* = 24) of clinicians felt that there is a lack of guidance in the orthodontic management of traumatised teeth and pulpal sequelae.

**Conclusion:**

Orthodontists are not following a standardised protocol in their examination of teeth with a history of trauma prior to orthodontic treatment. There is a need within the orthodontic specialty to create a standardised protocol to assess teeth with a history of dental trauma.

**Supplementary Information:**

The online version contains supplementary material available at 10.1186/s12903-024-05286-3.

## Background

Dental trauma is an injury commonly seen in young children. Traumatic dental injuries (TDI) are often unnoticed, despite their relatively high prevalence and their significant impact on the individual and their family. According to a UK national survey in 2013, the prevalence of dental trauma to permanent incisors of children in all age groups was 9.1%, with boys experiencing injuries more frequently than girls. The incidence varied significantly across different age groups with the highest prevalence in 12-year-old at 12.4% [1, 2]. A link has been suggested between malocclusion and the incidence of TDI, with an increased overjet and incompetent lips being significant risk factors [3, 4]. Children are twice as likely to sustain a dental injury with an overjet of > 5 mm compared to a normal overjet of < 5 mm. These patients should be treated and educated on dental trauma prevention from an early age as well as encouraged to use mouthguards [5]. Orthodontic therapy has been suggested as a preventative measure in correcting unfavourable malocclusions and potentially avoiding TDIs [6]. Implementing early orthodontic treatment in young children, followed by a subsequent phase during early adolescence, may be more effective in reducing incisal trauma in patients with prominent upper incisors compared to a single course of treatment administered during adolescence [7, 8].

Literature has shown that as many as 1 in 10 patients referred to an orthodontist had a history of dental trauma prior to active orthodontic treatment [4, 9]. Traumatised teeth are at greater risk of developing complications during orthodontic treatment such as external root resorption, invasive cervical resorption, pulpal necrosis, and pulp canal obliteration. Therefore, it is critical to record a dental trauma history prior to commencing orthodontic treatment to enable evaluation of potential complications and alter treatment planning if necessary [10]. Evidence from retrospective cohort studies has shown that teeth experiencing trauma during active orthodontic treatment have a significantly greater risk of developing pulpal necrosis compared to traumatised teeth without orthodontic intervention [11]. Moreover, teeth that have experienced extrusion, intrusion or lateral luxation injuries were significantly more likely to suffer from pulpal necrosis during orthodontic treatment compared to teeth experiencing trauma without orthodontic intervention [11].

Within current orthodontic literature, there is a scarcity of orthodontic research investigating the treatment planning of patients with a history of dental trauma. Traditionally, methods to examine orthodontists’ knowledge and management of patients with a history of dental trauma consisted of surveys with closed questions. The aim of this study was to explore whether orthodontists consider endodontic implications when managing traumatised teeth and gain an understanding of their opinions, as well as their treatment planning strategies for hypothetical cases based on vignette surveys.

## Methods

A cross-sectional vignette survey was distributed to UK-registered specialist orthodontists electronically over a 4-month period. The research obtained ethical approval from the ethics committee at the University of Liverpool (ID 3002). The purpose of the survey was to evaluate orthodontic specialists’ and consultants’ experience in the management of patients with a history of dental trauma. General dental practitioners (GDP), orthodontic specialist trainees, all other dental specialities and incomplete surveys were excluded from the study as they did not meet the inclusion criterion of being UK-registered specialist orthodontists.

Specialist orthodontists were recruited for participation using electronic means and social media platforms. An invitation link was distributed on Facebook and emailed to orthodontists. Both public and private dental groups were contacted to reach out to as many orthodontic specialists as possible. Orthodontists within Liverpool University Dental Hospital were contacted via email with a link to the survey. They also kindly shared this link with fellow orthodontic colleagues in other units, through snowball sampling. The questionnaire was constructed using ‘Select Survey’ software (ClassApps, (United States)). Recruitment was repeated twice monthly to maximise survey uptake Consent was gained from the participants, who were also informed that the data collected was anonymous.

The survey consisted of three parts (see Supplemental file 1). Parts one and two involved multiple-choice questions exploring the demographic data and participants’ previous experience of treating previously traumatised teeth (Table 1).

**Table 1:** Summary of the themes explored in part one and two of the survey

**Demographic data** **•** Participant’s: gender, region of work, duration of orthodontic career, primary role, main work setting (hospital/ private practice) and involvement in teaching
**Dental trauma experience and exposure**: **•** Level of confidence when dealing with traumatised teeth and number of dental trauma cases seen in the last 12 months
**Pre-treatment examination of a traumatised tooth**: **•** Appropriate radiographs and sensibility testing
**Participant’s knowledge on the available guidelines**: **•** Guidelines relating to orthodontic treatment of traumatised teeth

The third part of the survey involved three clinical case vignettes with the aim to explore the orthodontists’ understanding of endodontic risk with the provision of orthodontic treatment. The cases included a variety of traumatic complications including a mid-root fracture, pulp canal obliteration and an immature non-vital central incisor. The questions focused on choice of further investigations of the tooth and the appropriate time to start orthodontic treatment post dental trauma (Table 2).

The survey was piloted prior to the distribution by three orthodontic specialists based at Liverpool University Dental Hospital and one orthodontist who worked in an NHS orthodontic practice. Amendments were made to the survey using feedback received to ensure it was succinct and delivered to its intended purpose.

The vignettes were analysed using thematic analysis. The data collected from the ‘Select Survey’ software was uploaded as a Microsoft Excel spreadsheet and ‘NVIVO 12’ application (Lumivero, (United States)), to allow further analysis of the data and to modify the themes and subthemes.

**Table 2:** Summary of the three vignette cases

	Case summary	Questions presented following the cases?
Vignette 1	A 12-year-old boy, fit and well with no known medical problems, attends the Orthodontist with his parents having fallen onto the handlebars of his scooter 2 weeks ago. He is suffering from tenderness and bleeding around the gingiva of the UR1 and UL1, he has tenderness on tapping his teeth with some mobility on both teeth. The patient is due to start orthodontic treatment for moderate upper arch crowding. On taking a periapical radiograph, you suspect a mid-root fracture to the UL1 and UR1 respectively	• What other radiograph would you ask for other than a PA in this instance? • When would you start the orthodontic treatment? • Would you give the patient and parents any specific warning or advice prior to starting orthodontic treatment?
Vignette 2	A 13-year-old boy, fit and well with no known allergies, accidentally traumatised his tooth whilst playing sport 3 years ago. He presents to your orthodontic clinic with discolouration of the UL1. The patient has a Class 2 Division 1 incisor relationship with an 8 mm overjet. The patient’s mother is concerned with the yellow discolouration of this tooth. Otherwise, the tooth is asymptomatic. A periapical radiograph of the UL1 shows a completely obliterated canal. The patient is about to start orthodontic course of treatment	• What sensibility testing would you carry out on this tooth? • Orthodontic treatment is planned for this patient, how would this change your management? Would you do anything differently and what warnings would you give the patient? • Having discussed the findings with the patient and his parents, when would you start the orthodontic treatment for this patient?
Vignette 3	A 12-year-old patient, otherwise fit and well, had a fall whilst playing with her friends in the playground 4 years ago. The patient’s mother is concerned about the gradual discolouration associated with the UR1. The patient has no other symptoms. The tooth is not mobile and has no localised deep pocketing. She is due to start orthodontic treatment and on taking a periapical radiograph, you diagnose an immature UR1 with open apex	• Sensibility testing of the UR1 was deemed unreliable, would you consider root canal treat for this tooth or wait for further root development? And why? • If you feel root end closure is required for the UR1, who would you refer this patient for further treatment and could you explain your choice? • How soon, following root end closure, would you begin orthodontic treatment? • Would this injury and subsequent treatment affect your orthodontic management of the case? If so, how would it affect it? • Do you feel experienced/ confident to treat this case? • Are there guidelines for the orthodontic treatment of MTA root end closured teeth? If so, can you name it?

## Results

A total of 76 participants met the inclusion criteria, with the gender distribution comprising 55% (*n* = 42) males and 45% (*n* = 34) females. Of these, 36 were consultant orthodontists, 34 were orthodontic specialists and 6 were Post CCST trainees in orthodontics. The largest proportion 45% (*n* = 34) of respondents worked in England, with 34% (*n* = 26) of the respondents practising in Scotland, 12% (*n* = 9) in Northern Ireland and 9% (*n* = 7) in Wales. The distribution of professional experience among the orthodontists surveyed indicated that 28% (*n* = 21) had 0–5 years and 6–10 years of experience. Additionally, 20% (*n* = 15) of the respondents had been practising orthodontics for 21–30 years, and two participants had more than 30 years of experience.

**Fig. 1:**
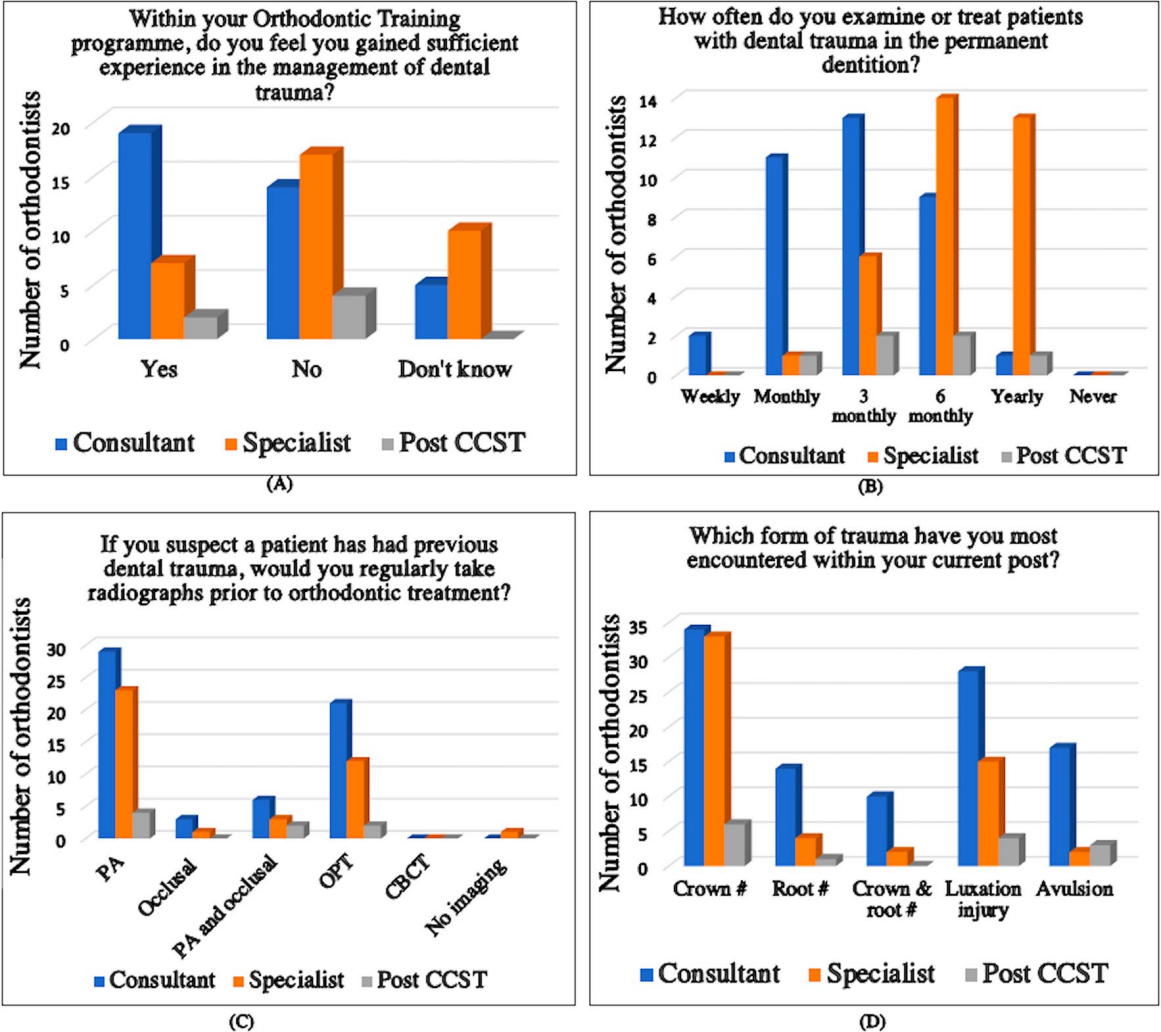
Results showing answers to part two of the questionnaire

The participants were asked about their exposure to dental trauma throughout their years of orthodontic specialist training. Over half of the consultants 53% (*n* = 19) felt they had sufficient experience of dental trauma throughout their training compared to 21% (*n* = 7) of specialist orthodontists and 33% (*n* = 2) of post-CCST trainees (Fig. 1A).

The results showed that consultants reported seeing greater numbers of trauma patients on a monthly and 3 monthly basis, compared to specialists and post CCSTs. The survey shows that all grades of specialists deal with patients with acute or past trauma (Fig. 1B). The survey results revealed that 75% (*n* = 27) of consultants expressed confidence in managing patients with dental trauma, compared to only 35% (*n* = 12) of the specialists. Most specialists reported treating a very low number of patients (< 5%) who sustained trauma compared to consultants.

The survey investigated the range of diagnostic procedures orthodontists would perform on a traumatized tooth. Most consultants (80%, *n* = 29) would take a periapical (PA) radiograph to examine a tooth they suspected of having trauma with the next favourable choice (58%, *n* = 21) being an orthopantomogram (OPT). Few consultants (8%, *n* = 3) and specialists (3%, *n* = 1) would take a stand-alone occlusal oblique radiograph (Fig. 1C). More than half of post CCSTs (67%, *n* = 4) and one third (33%, *n* = 12) of consultants reported that they do not carry out any form of sensibility testing when assessing a traumatised tooth. Consultants were also likely to treat more root fractures, crown root fractures and luxation injuries than specialists (Fig. 1D).

Participants’ responses revealed that 56% (*n* = 19) of orthodontic specialists were unaware of or lacked knowledge of a local trauma service. All post-CCSTs, 83% (*n* = 30) of consultants, and 47% (*n* = 16) of specialists reported familiarity with at least one guideline, specifically mentioning those by Kindelan and Day, as well as the International Association of Dental Traumatology (IADT) [3, 10].

Part 3 of the survey was the Vignette analysis.

### Vignette one

**Fig. 2:**
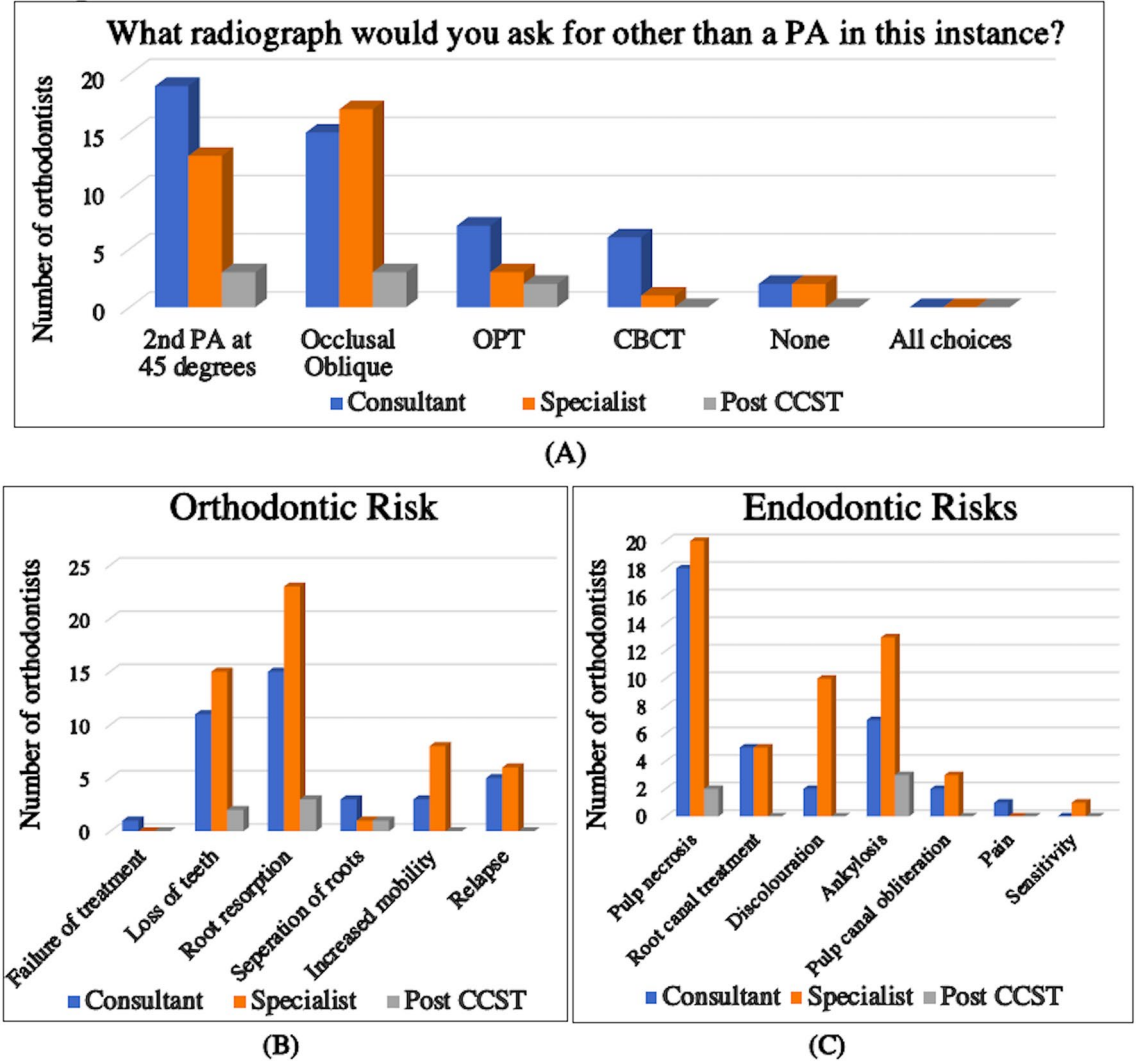
Charts summarising the participant’s responses to vignette one

This vignette presented a patient with a mid-root fracture affecting the UL1 and UR1. Almost half the number of respondents would consider taking an occlusal oblique radiograph to examine for a root fracture (43%, *n* = 33). Few consultants (17%, *n* = 6) would request a CBCT while 5% (*n* = 2) of consultants and 6% (*n* = 2) of specialists would take no radiographs at all for this presentation (Fig. 2A). Less than half of the consultants (44%, *n* = 16) and specialists (38%, *n* = 13) would refer this case for a second opinion to a multidisciplinary team (MDT) clinic, whilst 67% (*n* = 4) of Post CCSTs would monitor for 12–24 months prior to treatment. In addition, 21% (*n* = 7) of specialists recorded they would not attempt the treatment.

For the open-ended question, participants were asked to report specific risks they would warn patients prior to starting treatment. The most common risks highlighted were pulp necrosis, loss of teeth and root resorption (Fig. 2B and C).

### Vignette two

**Fig. 3:**
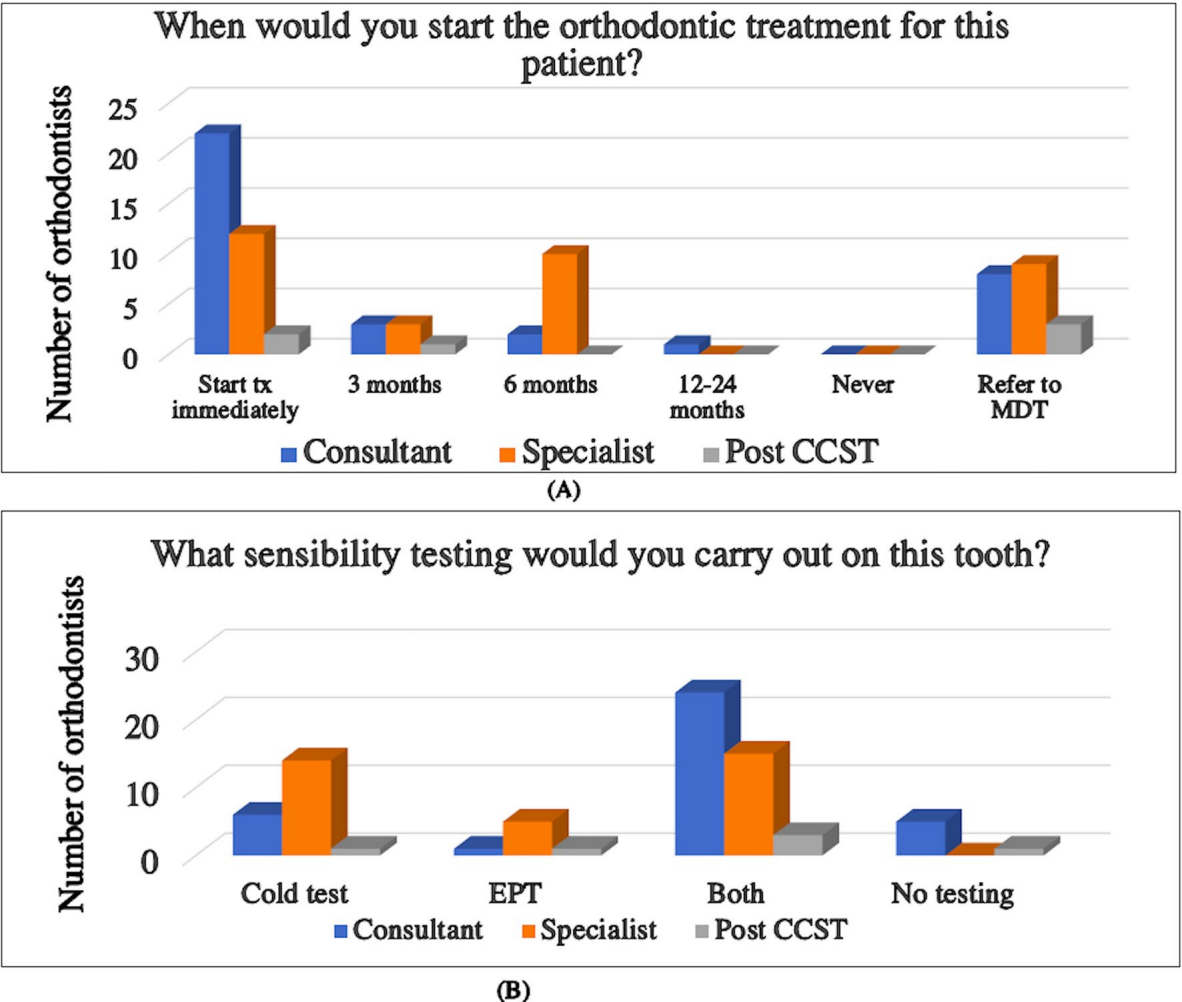
Charts summarising the participant’s responses to vignette two

This vignette presented an UL1 with pulp canal obliteration following previous trauma. It explored the choices of sensibility testing which were most appropriate for the tooth with 67% (*n* = 24) of consultants, 50% (*n* = 3) of post CCSTs and 44% (*n* = 15) of specialists reporting that would carry out cold testing and electric pulp testing (EPT) (Fig. 3B). However, 17% (*n* = 1) of post-CSSTs and 14% (*n* = 5) of consultants wouldn’t carry out any sensibility testing at all. Most consultants (61%, *n* = 22) would begin treatment immediately in this case compared to over a third of specialists (36%, *n* = 12) and Post CCSTs (33%, *n* = 2) (Fig. 3A).

### Vignette three

**Fig. 4:**
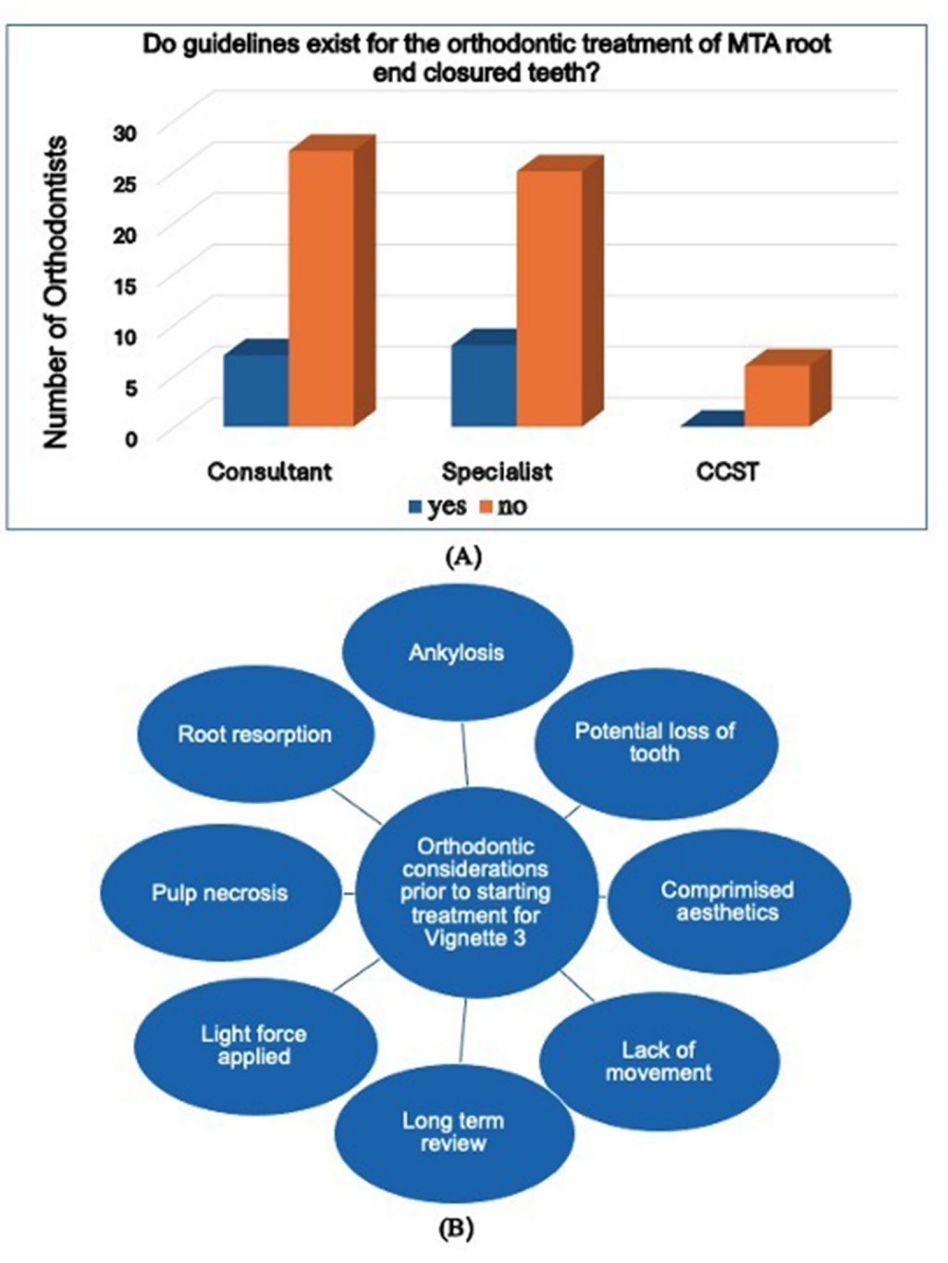
Summary of the participant’s responses to case three

Participants were presented with a discoloured traumatised tooth which had an immature apex. In this case, 56% (*n* = 42) of the participants believed that the tooth is non-vital based on the patient’s age, stage of root development and discolouration. Consequently, 39% (*n* = 14) of consultants, 29% (*n* = 10) of specialists and 33% (*n* = 2) of post-CCSTs would wait at least 6 months after root end closure before proceeding with the orthodontic treatment. 17% (*n* = 6) of consultants and 21% (*n* = 7) of specialists would refer the patient for a second opinion before starting treatment (Fig. 4B). Less than half (46%, *n* = 35) of orthodontists indicated that their treatment approach would remain unchanged, yet they committed to routinely evaluating the tooth both clinically and radiographically during active orthodontic treatment.

Most participants (77.6%, *n* = 59) felt there was no current literature or evidence to help them decide on the best treatment option for a tooth presenting with an open apex. Figure 4B summarises the clinical factors reported by the participants related to the orthodontic movement of an immature UR1 with a guarded prognosis. Additionally, 22% (*n* = 17) of the respondents cited Kindelan and Day, 2008, IADT, BSPD and Textbook of Dental Traumatology as presenting current evidence and guidance on this subject matter [3, 12, 13].

## Discussion

There is a scarcity of vignette-based surveys within orthodontics, with only a few studies exploring orthodontists’ knowledge of dental trauma and orthodontic management of traumatised teeth [14, 15]. This study achieved 76 responses which represents 5.5% of the total number of specialist orthodontists registered in the UK [16]. The gender distribution of 55% (*n* = 42) male and 45% female (*n* = 34) was recorded, deviating slightly from the General Dental Council’s (GDC) registry figures of 47.5% male and 52.5% female. Despite this discrepancy, the authors suggest that the study offers a reasonable representation of the UK orthodontist population.

The study’s results showed that orthodontic consultants reported seeing a greater volume of traumatised teeth needing orthodontic consultation, therefore gaining further confidence in managing cases involving dental trauma compared to orthodontic specialists. These results are unsurprising as orthodontic consultants usually undergo further training post specialisation to treat patients with more complex orthodontic treatment needs within secondary and tertiary care. In contrast, orthodontic specialists working in primary care settings usually see fewer complex cases. This can inadvertently lead to possible de-skilling of specialist orthodontists who may decide to treat trauma patients less frequently due to lack of confidence and training, and thus refer to orthodontic consultants. Sandler et al., found that UK orthodontists refer their cases to consultants due to lack of guidelines (11.4%), lack of experience (11%), lack of training (8.1%), and fear of litigation (0.47%) [14].

The frequency and number of cases with current or previous trauma under the care of participants differed between the grade of orthodontists. Most orthodontists examined a patient with a history of trauma every 3 or 6 months 28% (*n* = 21), and 33% (*n* = 25). The findings in this study were comparable findings of Sandler et al. who reported the frequency of trauma in specialist practice to be 38.6% at 3 months and 33.3% at 6 months [14].

Surprisingly, the results of this survey showed that 70% (*n* = 24) of specialists, 67% (*n* = 4) of Post CCSTs and 33% (*n* = 12) of consultants do not carry out any form of sensibility testing for a traumatised tooth. This is despite the guidelines from the IADT and American association of Endodontists (AAE) recommending sensibility testing as part of the management of traumatised teeth [17, 18]. Owtad et al. states that it’s important to perform a pulp test and take a periapical radiograph of traumatised teeth every 3–6 months to assess the condition of the periapical tissues during the orthodontic treatment [19]. This highlights that there may be an inconsistency in the trauma knowledge and teaching present among orthodontists. This is further reinforced by the responses obtained from the survey which stated that 53% (*n* = 19) of consultants felt they had sufficient experience of dental trauma throughout their training, compared to 21% (*n* = 7) of specialist orthodontists and 33% (*n* = 2) post CCST trainees.

The first vignette scenario demonstrated a 12-year-old boy with horizontal root fractures associated with the UL1 and the UR1 respectively. At present, there is limited guidance for the correct imaging choice for a horizontal root fracture. The IADT suggests a standard PA radiograph should be able to demonstrate a coronal third fracture. However, due to the oblique nature of the horizontal fractures in the mid and apical third of the root, an occlusal oblique may help to locate and diagnose the fracture more accurately [17]. An OPT was the choice of radiograph for around 16% (*n* = 12) of all orthodontists. Whilst still a commonly used radiograph for trauma assessment, its accuracy is less than that of a PA or occlusal oblique due to superimposition of the radiation beam which may produce artefacts resulting in poor definition to be able to examine for a fracture correctly [20].

The second vignette scenario asked orthodontists about their experience with regards to the treatment planning of UL1 which had become discoloured after trauma with a diagnosis of pulp canal obliteration (PCO). Most participants here would carry out both EPT and cold testing sensibility tests. Literature suggests that teeth which undergo periodontal ligament injury tend to have delayed response rates to sensibility testing for up to nine months and sometimes longer after the initial stimulus [21]. With regards to PCO, it is generally accepted that sensibility tests are unreliable with a progressive decrease in response rates to both thermal and EPT as the PCO becomes more pronounced [22]. However, teeth with partial PCO showed a greater response to electric pulp testing than teeth with complete PCO. Caution must be taken not to mistake a negative thermal or electrical pulp test for a non-vital pulp and other signs and radiographic images must be taken to collate further evidence of pulp necrosis [21]. A tooth with PCO due to trauma must be monitored throughout the orthodontic treatment and only a light force (less than 70 g pressure) should be applied to the tooth [23]. Kindelan and Day discussed the appropriate monitoring period for traumatised teeth, with a three-month monitoring period being suggested for minor injuries and 6–12 months for more severe injuries.

The third vignette study explored orthodontists’ treatment planning of a suspected non-vital UR1, which was otherwise asymptomatic with gradual grey discolouration and an immature apex. Opinions on treatment varied between clinicians with many orthodontists believing the tooth is non-vital due to the age of the patient and expected stage of root development. When examining the development of permanent central incisors, calcification begins at 3–4 months, the crown forms by 4–5 years and the root is completely developed by 9–10 years of age. Given the patient is 12 years old, it is unlikely that the tooth will continue to further develop [24], and therefore the majority of orthodontists’ diagnosis of pulp necrosis are logical. However, this diagnosis was made purely based on the patient’s age, expected root development, root closure age and discolouration of the crown; none of the written responses eluded to the utilisation of sensibility tests to confirm the diagnosis or having at least 2 signs to justify root canal treatment [25]. When looking at the monitoring time prior to starting orthodontic treatment, most clinicians would wait 6 months after root-end closure before proceeding with treatment. Sandler et al., discusses the orthodontic consideration for a non-vital tooth managed by Regenerative Endodontic Treatment (RET) [23]. However, there were no considerations for non-vital immature apices treated with apexification.

The orthodontic movement of teeth with an open apex has been poorly studied in literature. Root resorption following orthodontic treatment may be influenced by factors such as prolonged treatment duration, excessive force of application and the degree of apical movement [26]. A systematic review by Wasserman-Milhem [27] found that immature teeth underwent less root resorption, yet if the duration and force of treatment is high and long, then their risk is comparable to teeth with closed apices. Secondly, starting orthodontic treatment before full apical closure in immature teeth may reduce root resorption risk. However, confounding factors such as genetics, systemic disease, allergy, trauma, or habits were not considered [27].

### Study limitations

Due to the introduction of new legislation regarding the General Data Protection Regulation (GDPR) during the study, the original plan of contacting all orthodontic practitioners by acquiring their details through the General Dental Council (GDC) was not possible. Therefore, social media was selected as the main outlet to contact orthodontists. This method was very effective in collecting data. However, it excluded dentists with limited computer access and less familiarity with social media based dental professional groups. Consequently, only two respondents were highly experienced dentists with over 30 years post- qualification. The eligible completion rate of the survey was 58.5%, with 76 registered orthodontists completing the survey out of 130 who initiated it. To enhance the survey’s representativeness, a postal survey option could have been employed to reach a broader group of practitioners. Furthermore, the authors of any future studies may collaborate with the British Orthodontic Society for the survey distribution to improve the response rate.

The study focused on UK-registered orthodontic specialists who practice in the UK. The participant pool had nearly equal numbers of males and females by chance, but there was no control over variables like gender, race, age, city, university of training, or practice setting due to the web-based nature of the survey. This lack of control might have affected the generalizability of the results, as evidenced by the higher number of consultant orthodontists compared to specialist orthodontists in the study. A larger sample size could have helped mitigate the impact of uneven representation and improve the generalizability of the results.

With regards to the vignette study, the average time to complete the survey was less than 7.5 min, however, the cover page stated it may take up to 15 min, this may have potentially dissuaded some orthodontists from completing the survey due to time constraints, perceived effort required to complete the survey, boredom, lack of interest or having a busy schedule and unable to free time to complete the survey. Therefore, any future prospective studies may design a shorter survey to encourage more responses. This could be achieved by reducing the description of the vignette scenarios. A reward could also be offered to participants who complete the survey.

The authors suggest that any future prospective studies contain a qualitative component, such as incorporating one-on-one interviews or focus groups, to assess orthodontists’ confidence in managing traumatized teeth. This method would aim to identify in depth potential barriers regarding this issue.

## Conclusion

The effect of orthodontic movement on traumatised teeth and its impact on orthodontists’ treatment planning is a key concern shared amongst many orthodontists. A core finding was that post CCST trainees and orthodontists working in specialist practice felt that their orthodontic training lacked trauma management and experience to investigate the clinical significance of orthodontic therapy on traumatised teeth. Further research is required to evaluate the true extent and effect of orthodontics on teeth with a history of dental trauma, as well as the long-term outcomes for the teeth and the individuals it affected.

## Electronic supplementary material

Below is the link to the electronic supplementary material.

Supplementary Material 1

## Data Availability

The datasets supporting the conclusions of this article are available at the University of Liverpool Repository, [https://livrepository.liverpool.ac.uk/3073672/].

## Abbreviations

TDI: Traumatic dental injuries
GDP: General dental practitioners
NHS: National health service
GDC: General Dental Council
GDPR: General Data Protection Regulation
OPT: Orthopantomogram
PA: Periapical Radiograph
CBCT: Cone beam computed tomography
Post CCST: Certificate of completion of speciality training
