# Supplementary figures and images for: Do orthodontists consider endodontic complications in their orthodontic management of teeth with a history of dental trauma? A vignette survey
Source: BMC Oral Health. 2025 Jan 16;25:80. doi: 10.1186/s12903-024-05286-3 (PMC11740483; doi:10.1186/s12903-024-05286-3)

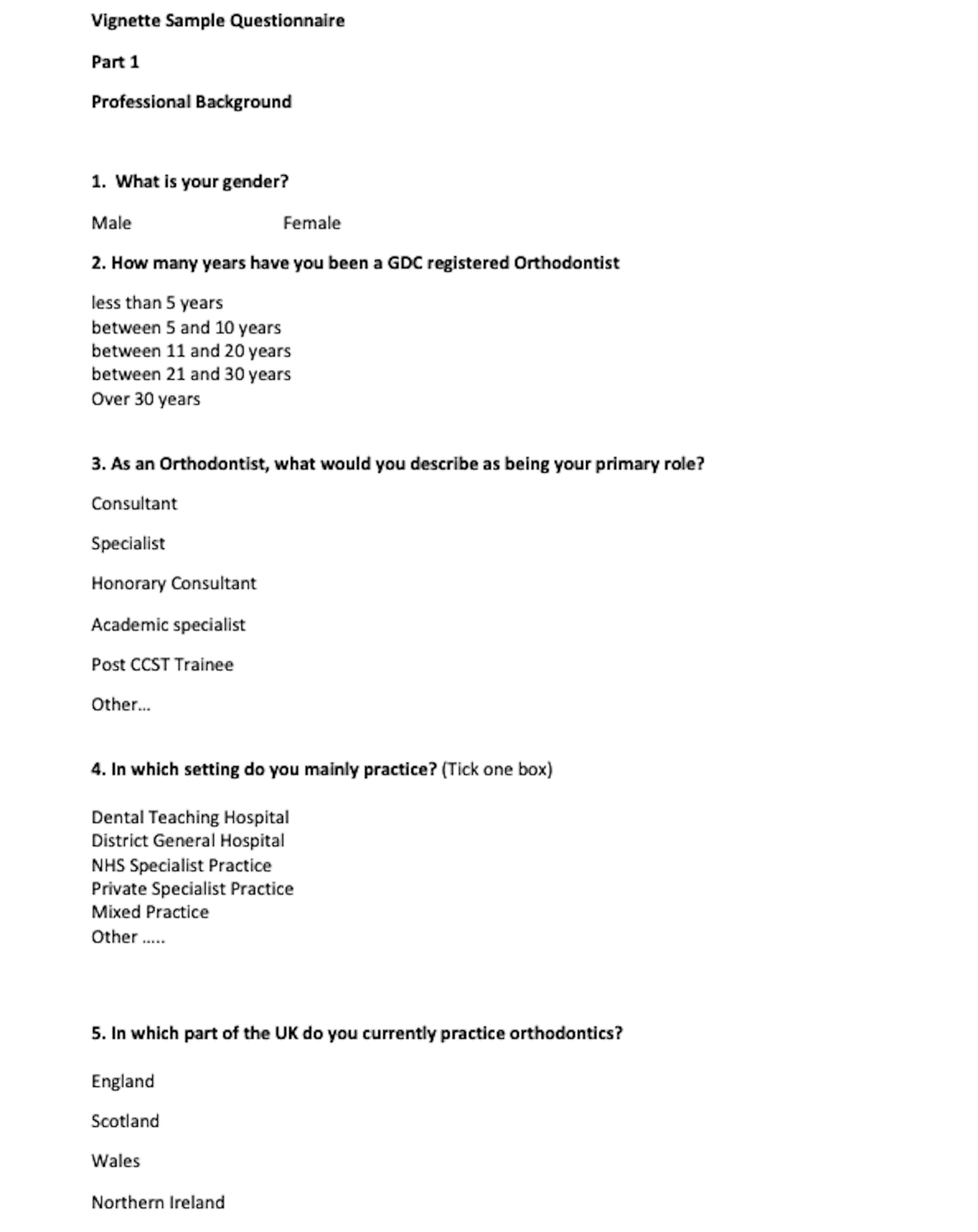


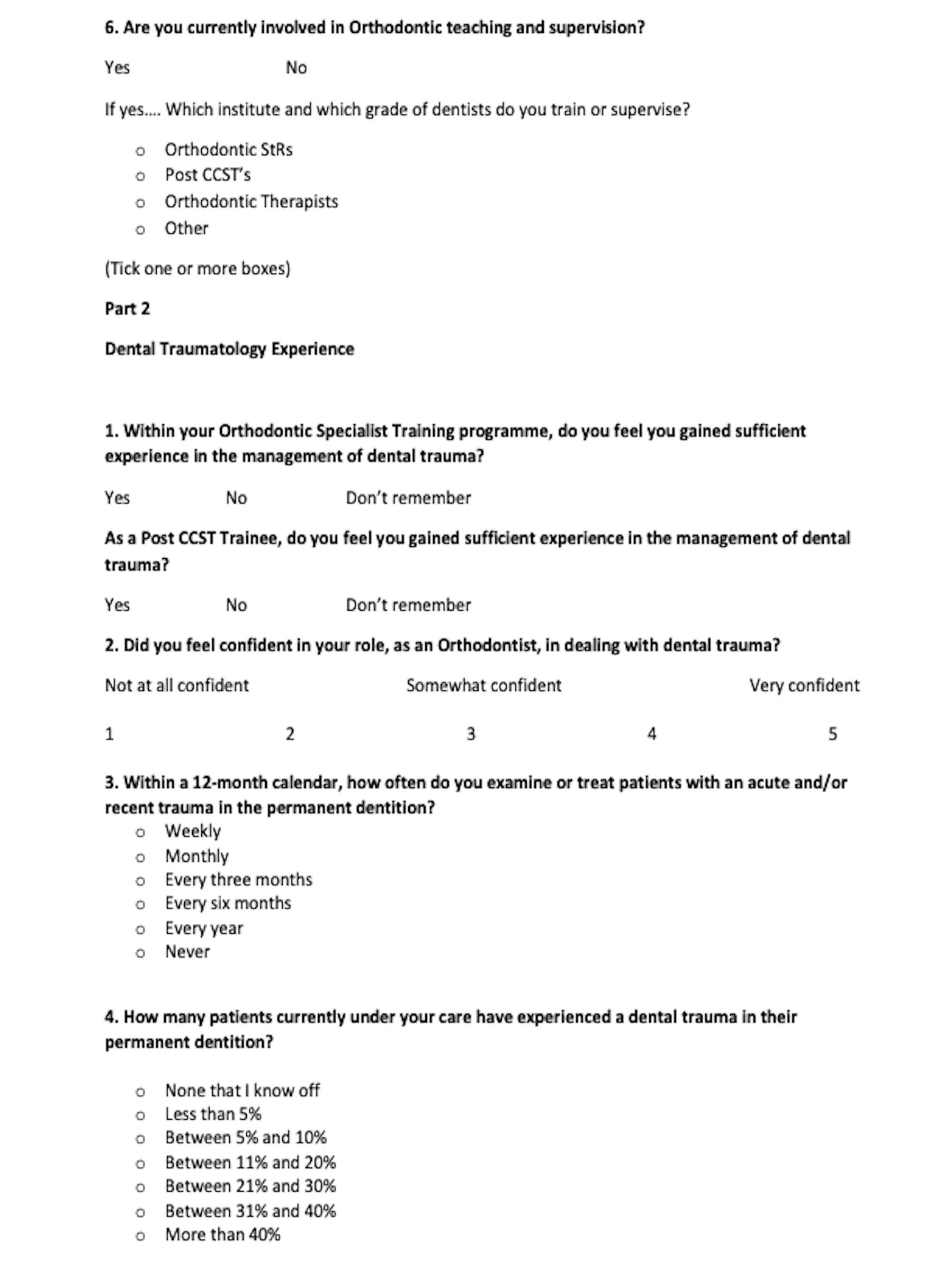


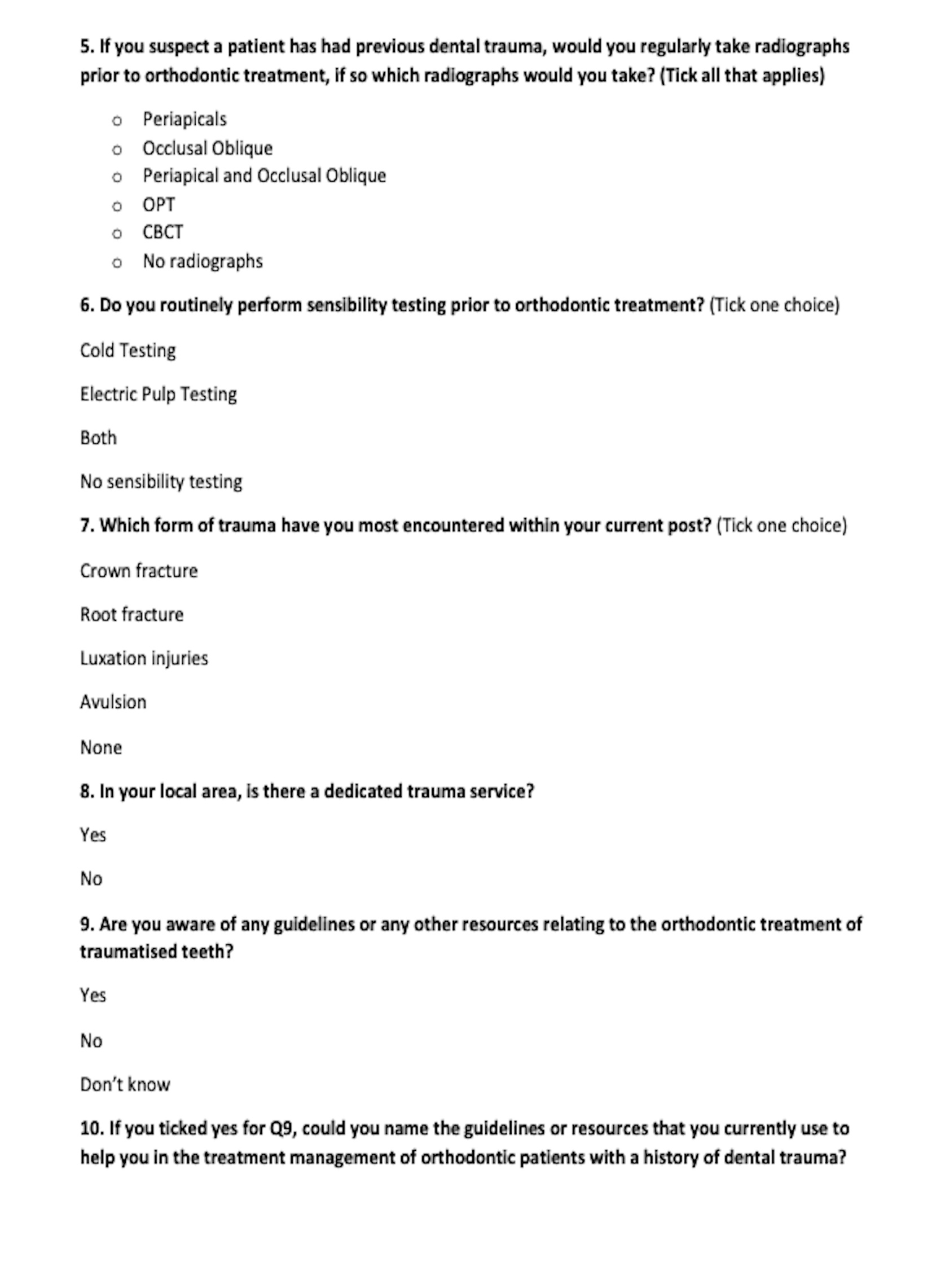


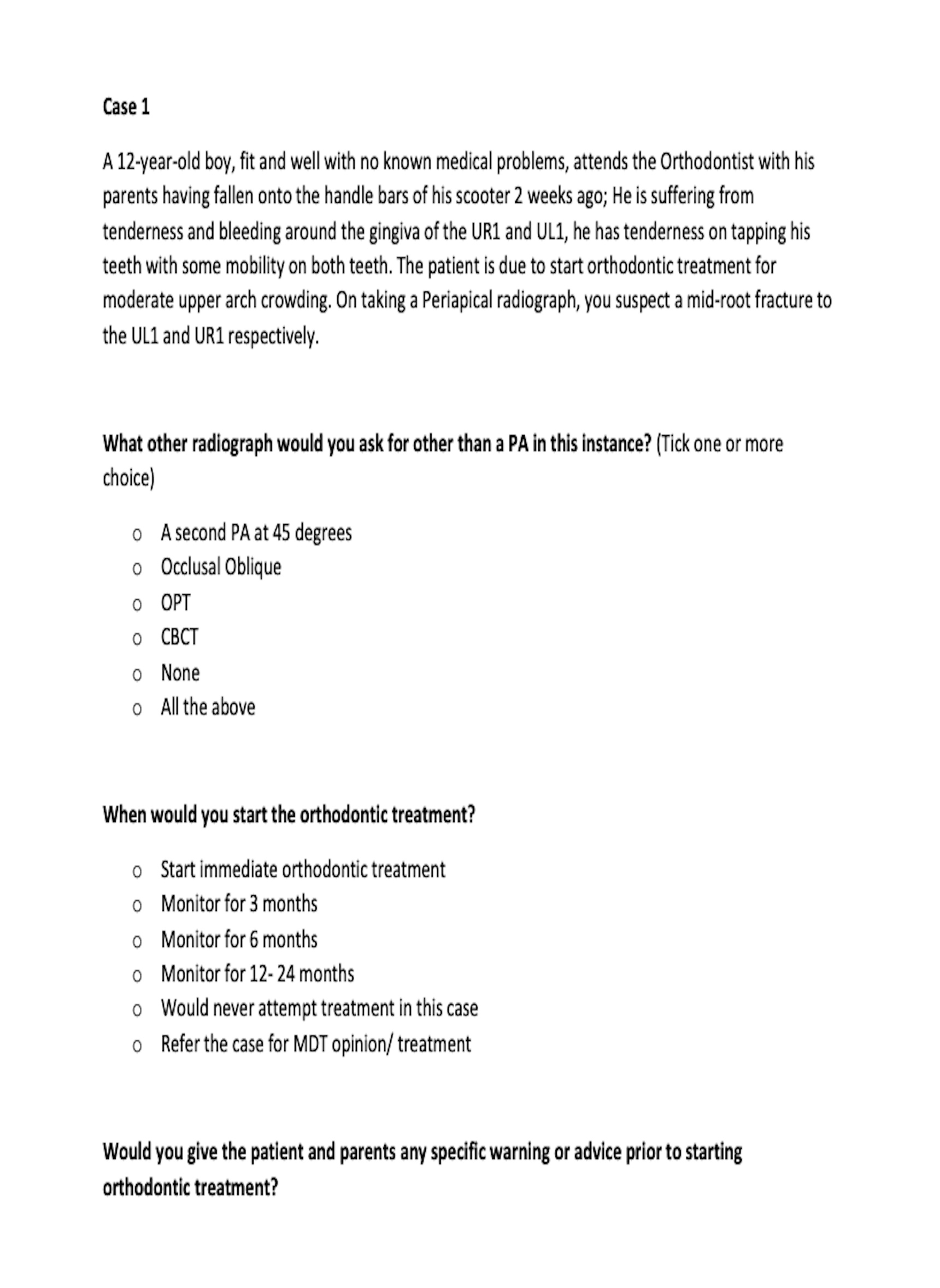


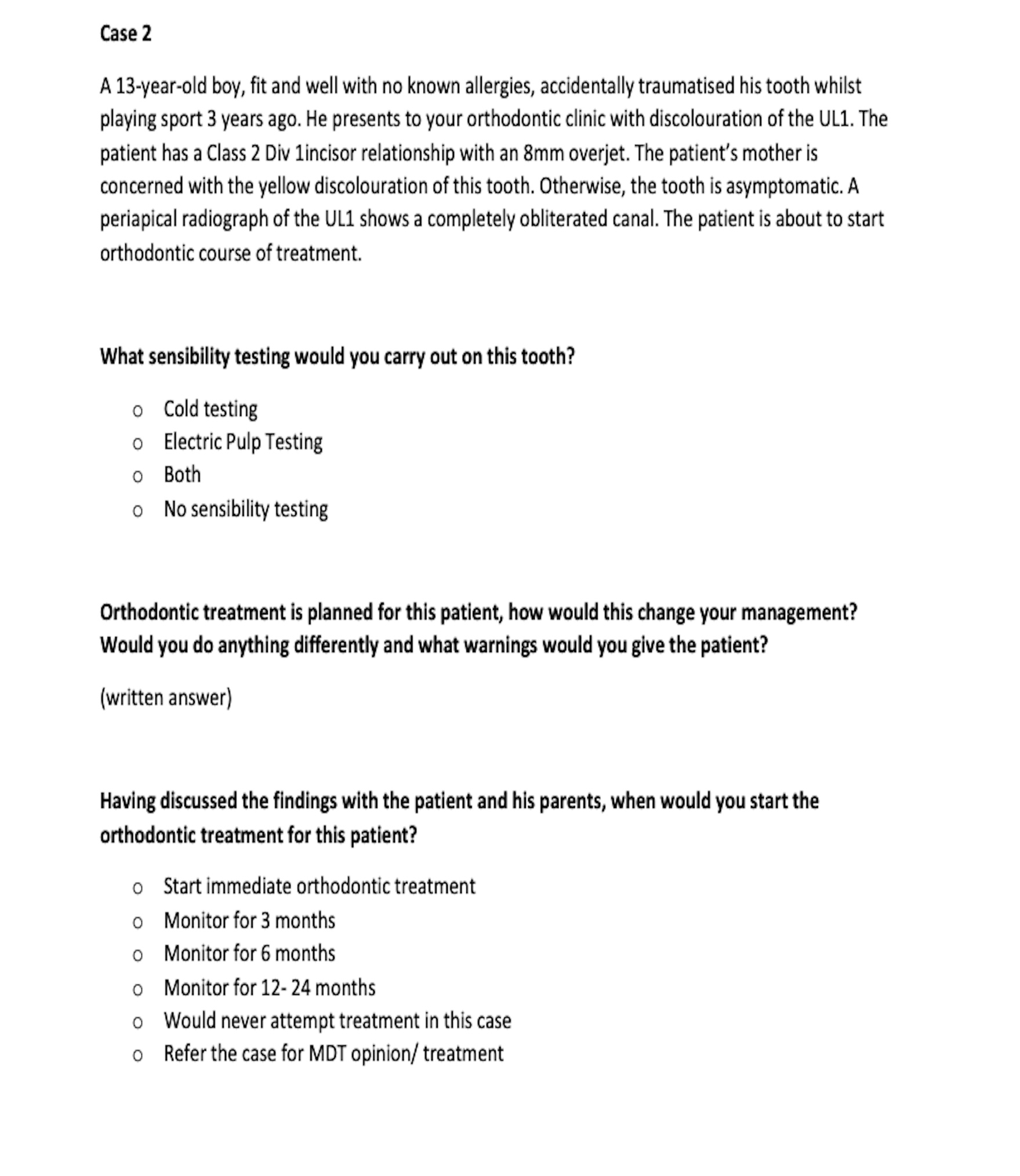


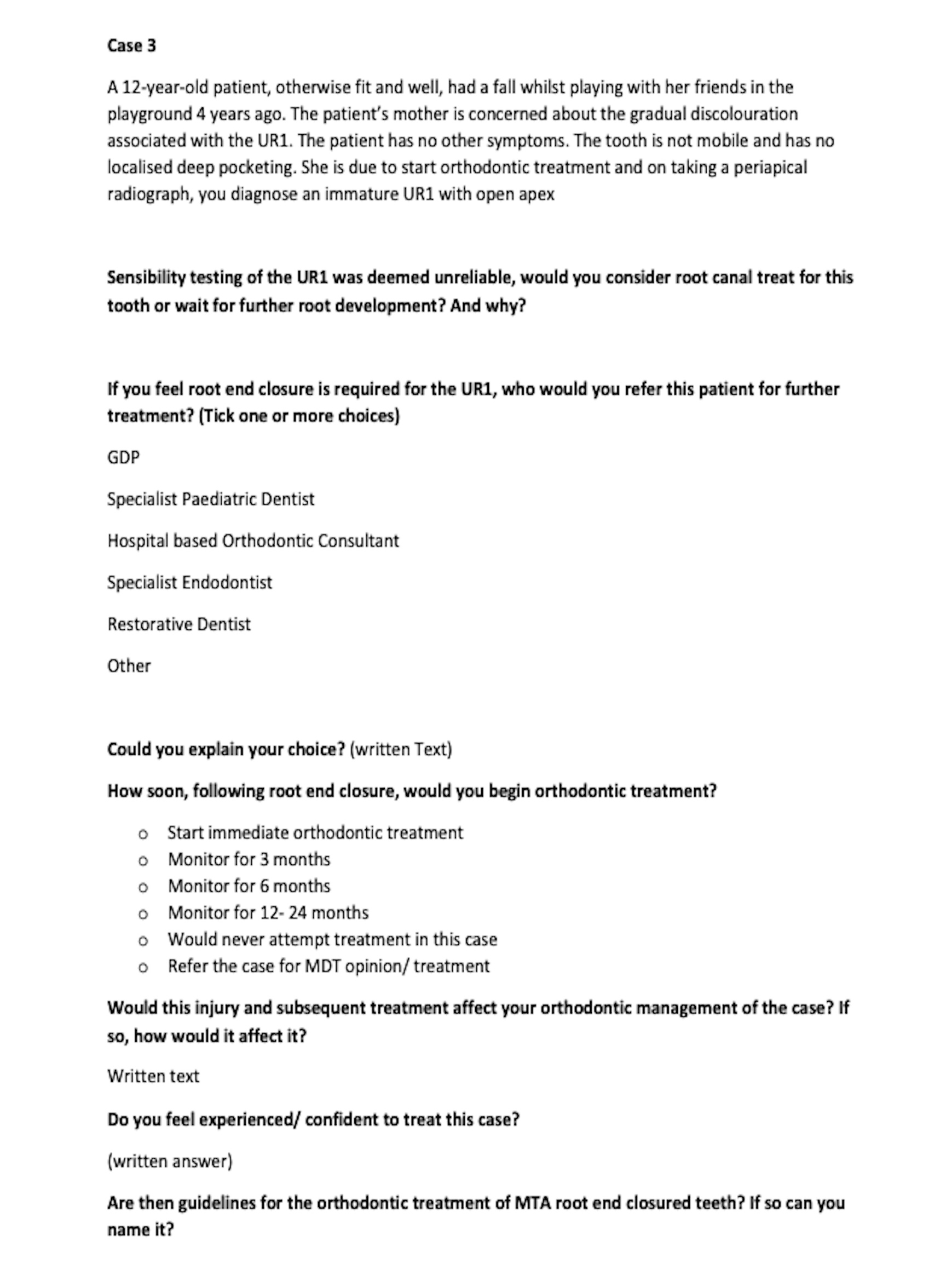

Supplement: Supplementary file 1 — Supplementary Material 1 [file 12903_2024_5286_MOESM1_ESM.docx]
